#  Genome sequences published outside of *Standards in Genomic Sciences,* October - November 2012

**DOI:** 10.4056/sigs.3597227

**Published:** 2012-12-12

**Authors:** Oranmiyan W. Nelson, George M. Garrity

**Affiliations:** 1 Editorial Office, Standards in Genomic Sciences and Department of Microbiology, Michigan State University, East Lansing, MI, USA

## Abstract

The purpose of this table is to provide the community with a citable record of publications of ongoing genome sequencing projects that have led to a publication in the scientific literature. While our goal is to make the list complete, there is no guarantee that we may have omitted one or more publications appearing in this time frame. Readers and authors who wish to have publications added to subsequent versions of this list are invited to provide the bibliographic data for such references to the SIGS editorial office.

## Domain*Archaea*

### Phylum *Crenarchaeota*

“*Thermogladius cellulolyticus*” 1633, sequence accession CP003531 [1]

### Phylum *Euryarchaeota*

*Methanomassiliicoccus luminyensis*, sequence accession CAJE01000001 through CAJE01000026 [2]
*Pyrococcus sp.* Strain ST04, sequence accession CP003534 [3]

## Domain*Bacteria*

### Phylum *Nitrospirae*

*Leptospirillum ferrooxidans* Strain C2-3, sequence accession AP012342 [4]

### Phylum *Cyanobacteria*

Prochlorococcus marinus MED4, sequence accession BX548174 [5]

### Phylum *Proteobacteria*

*Acinetobacter sp.* Strain HA, sequence accession AJXD00000000 [6]
*Acinetobacter venetianus* RAG-1^T^, sequence accession AKIQ00000000 [7]
*Aeromonas aquariorum*, sequence accession BAFL01000001 through BAFL01000036, and AP012343 [77]
*Agrobacterium tumefaciens* CCNWGS0286, sequence accession AGSM00000000 [8]
*Alcaligenes faecalis subsp. faecalis* NCIB 8687, sequence accession AKMR01000001 through AKMR01000186 [9]
*Alishewanella aestuarii* Strain B11^T^, sequence accession ALAB00000000 [10]
*Alishewanella agri* BL06^T^, sequence accession AKKU00000000 [11]
*Bartonella birtlesii* strain IBS 135^T^, sequence accession AKIP00000000 [12]
*Brucella abortus* A13334, sequence accession CP003176.1 (Chromosome I), CP003177.1 (Chromosome II) [13]
*Brucella canis* Strain HSK A52141, sequence accession CP003174.1 (chromosome I), and CP003175.1 (chromosome II) [14]
*Brucella melitensis* 16M13w*,* sequence accession AHWE00000000 [15]
*Brucella melitensis* 16M1w*,* sequence accession AHWD00000000 [15]
*Brucella melitensis* S66, sequence accession AHWB00000000 [16]
*Brucella melitensis*16M*,* sequence accession AHWC00000000 [15]
*Burkholderia sp.* Strain KJ006, sequence accession CP003514 (chromosome I), CP003515 (chromosome II), CP003516 (chromosome III), and CP003517 (plasmid pKJ006) [17].
*Burkholderia terrae* Strain BS001, sequence accession AKAU00000000 [18]
*Burkholderia thailandensis* MSMB43, sequence accession AJXB00000000 [19]
*Candidatus* Sulfurovum sediminum, sequence accession AJLE00000000 [20]
*Catenovulum agarivorans* YM01^T^, sequence accession AJWM00000000 [21]
*Citrobacter sp.* Strain A1, sequence accession AKTT00000000 [22]
*Citreicella aestuarii* Strain 357, sequence accession AJKJ00000000 [86]
*Cronobacter sakazakii* ES15, sequence accession CP003312 [23]
*Dickeya zeae* Strain ZJU1202, sequence accession AJVN00000000 [24]
*Enterobacter cloacae* GS1, sequence accession AJXP00000000 [25]
*Enterobacter radicincitans* DSM16656^T^, sequence accession AKYD00000000 [26]
*Enterobacter sp.* Isolate Ag1, sequence accession AKXM00000000 [89]
*Escherichia coli* J53, sequnce accession AICK00000000 [28]
*Escherichia coli* LCT-EC106, sequence accession [29]
*Escherichia coli* NCCP15647, sequence accession AJMB00000000 [30]
*Escherichia coli* W26, sequence accession AGIA00000000 [31]
*Gluconobacter oxydans* WSH-003, sequence accession AHKI00000000 [32]
*Halomonas stevensii* S18214^T^, sequence accession AJTS00000000 [33]
*Helicobacter cinaedi* Strain PAGU611, sequence accession AP012344 (chromosome) and AP012345 (plasmid) [34]
*Helicobacter pylori* hpEurope Strain N6, sequence accession CAHX01000001 to CAHX01000054 [35]
*Herbaspirillum lusitanum* P6-12, sequence accession AJHH00000000 [36]
*Herbaspirillum* sp. Strain GW103, sequence accession AJVC00000000 [37]
*Hydrocarboniphaga effusa* strain AP103^T^, sequence accession AKGD00000000 [38]
*Hydrogenophaga* sp. Strain PBC, sequence accession AJWL00000000 [39]
*Klebsiella oxytoca* E718, sequence accession CP003683 [40]
*Methylobacterium extorquens* sp. strain 4-46, sequence accessions NC_010511, NC_010373, NC_010374 [41]
*Methylobacterium extorquens* strain BJ001, sequence accessions NC_010725, NC_010727, NC_010721 [41]
*Methylobacterium extorquens* strain CM4, sequence accessions NC_011757, NC_011758, NC_011760 [41]
*Methylobacterium extorquens* strain JCM 2831, sequence accessions NC_010510, NC_010509, NC_010514, NC_010517, NC_010518, NC_010502, NC_010504, NC_010507 [41]
*Methylobacterium extorquens* strain ORS 2060, sequence accessions NC_011894,NC_011892, NC_011887, NC_011893, NC_011895, NC_011888, NC_011889, NC_011890 [41]
*Methylobacterium extorquens* strain PA1, sequence accessions NC_010172 [41]
*Methylobacterium sp.* Strain GXF4, sequence accession AKFK00000000 [42]
*Methylophaga sp.* Strain JAM1, sequence accession CP003390 [43]
*Methylophaga sp.* Strain JAM7 sequence accession CP003380 (chromosome), CP003381 (plasmid) [43]
*Modestobacter marinus* Strain BC501, sequence accession FO203431 [44]
*Mycobacterium massiliense* M18, sequence accession AJSC00000000 [45]
*Neisseria meningitidis* Capsule Null Locus Strain, sequence accession CAJS01000001 through CAJS01000042 [46]
*Novosphingobium sp.* Strain Rr 2-17, sequence accession AKFJ00000000 [47]
*Providencia stuartii* Clinical Isolate MRSN 2154, sequence accession CP003488 [48]
*Pseudaminobacter salicylatoxidans* KCT001, sequence accession CAIU00000000 [49]
*Pseudoalteromonas issachenkonii* PAMC 22718, sequence accession AJTK00000000 [50]
*Pseudomonas aeruginosa* Strain SJTD-1, sequence accession AKCM00000000 [51]
*Pseudomonas aeruginosa* Strain XMG, sequence accession AJXX00000000 [52]
*Pseudomonas fuscovaginae* CB98818, sequence accession ALAQ00000000 [53]
*Pseudomonas pseudoalcaligenes* KF707, sequence accession AJMR00000000 [54]
*Pseudomonas putida* Strain ND6, sequence accession CP003588 [55]
*Pseudomonas putida* Strain SJTE-1, sequence accession AKCL00000000 [56]
*Pseudomonas sp.* Strain HYS, sequence accession AJJP00000000 [57]
*Pseudomonas sp.* Strain M47T1, sequence accession AJWX00000000 [58]
*Pseudomonas stutzeri* TS44, sequence accession AJXE00000000 [59]
*Pseudomonas stutzeri* Strain JM300, sequence accession CP003725 [103]
*Ralstonia sp.* strain PBA, sequence accession AJWL00000000 [60]
*Rhodanobacter* DSM 17631, sequence accession AJXT00000000 [61]
*Rhodanobacter* DSM 18449, sequence accession AJXU00000000 [61]
*Rhodanobacter* DSM 18863, sequence accession AJXW00000000 [61]
*Rhodanobacter* DSM 24678, sequence accession AJXV00000000 [61]
*Rhodanobacter* strain 115, sequence accession AJXS00000000 [61]
*Rhodanobacter* strain DSM 23569, sequence accession AGIL00000000 [61]
*Rickettsia australis* strain Phillips^T^, sequence accession AKVZ00000000 [62]
*Rickettsia conorii* subsp. caspia*, sequence accession AJUR00000000* [63]
*Rickettsia conorii* subsp. israelensis, sequence accession AJVP00000000 [64]
*Rickettsia sp.* Strain MEAM1, sequence accession AJWD00000000 [65]
*Salmonella enterica* serotype Newport CVM19443, sequence accession AHUB00000000 [66]
*Salmonella enterica* serotype Newport CVM19470, sequence accession AHUE00000000 [66]
*Salmonella enterica* serotype Newport CVM19593, sequence accession AHUD00000000 [66]
*Salmonella enterica* serotype Newport CVM21538, sequence accession AHTV00000000 [66]
*Salmonella enterica* serotype Newport CVM21550, sequence accession AHTT00000000 [66]
*Salmonella enterica* serotype Newport CVM33953, sequence accession AHTM00000000 [66]
*Salmonella enterica* serotype Newport CVM35185, sequence accession AHTJ00000000 [66]
*Salmonella enterica* serotype Newport CVM37978, sequence accession AHUC00000000 [66]
*Salmonella enterica* serovar Typhi UJ308A, sequence accession AJTD00000000 [67]
*Salmonella enterica* Serovar Typhi UJ816A, sequence accession AJTE00000000 [67]
*Serratia marcescens* strain LCT-SM213, seqeunce accession AJUV00000000 [68]
*Serratia plymuthica* Strain PRI-2C, sequence accession AJTB00000000 [69]
*Serratia sp.* Strain M24T3, sequence accession [70]
*Sinorhizobium fredii* USDA257, sequence accession CP003563 through CP003582 [71]
*Sphingobium indicum* B90A, sequence accession AJXQ00000000 [72]
*Stenotrophomonas maltophilia* PML168, sequence accession CAJH01000001 through CAJH01000097 [73]
*Sulfuricella denitrificans* skB26, sequence accession BAFJ01000001 through BAFJ01000023 [74]
*Xanthomonas campestris* JX, sequence accession AJVO00000000 [75]
*Yersinia pestis* Strain 2501, sequence accession AKVQ00000000 [76]

### Phylum Firmicutes

“*Geobacillus thermoglucosidans*” TNO-09.020, sequence accession AJJN00000000 [93]
*Aerococcus viridans* LL1, sequence accession AJTG00000000 [78]
*Bacillus anthracis* H9401, sequence accession CP002091.1 ( chromosome), CP002092.1.1 (plasmid pXO1), and CP002093.1 (plasmid pXO2) [79]
*Bacillus atrophaeus* C89, sequence accession AJRJ00000000 [80]
*Bacillus cereus* NC7401, sequence accession AP007209 (chromosome), AP007210 (plasmid pNCcld), AP007211 (plasmid pNC1, 48 kb), AP007212 (plasmid pNC2, 5 kb), AP007213 (plasmid pNC3, 4 kb), and AP007214 (plasmid pNC4, 3 kb) [81]
*Bacillus methanolicus* MGA3, sequence accession ADWW00000000 [82]
*Bacillus methanolicus* PB1, sequence accession AFEU00000000 [82]
*Bacillus siamensis* KCTC 13613^T^, sequence accession AJVF00000000 [83]
*Bacillus sp.* Strain 5B6, sequence accession AJST00000000 [84]
*Bacillus sp.* Strain 916, sequence accession AFSU00000000 [85]
*Clostridium beijerinckii* Strain G117, sequence acceession AKWA00000000 [87]
*Corynebacterium pseudotuberculosis* Strain 1/06-A, sequence accession CP003082 [88]
*Enterococcus faecalis* D32, sequence accession CP003726 through CP003728 [90]
*Enterococcus faecalis* strain NP-10011, sequence accession AB712291 [91]
*Enterococcus faecium* Clinical Isolate LCT-EF128, sequence accession AJUP00000000 [27]
*Enterococcus hirae* (*Streptococcus faecalis*) ATCC 9790, sequence accession CP003504 (chromosome), NC_015845 (plasmid pTG9790) [92]
*Lactobacillus mucosae* LM1, sequence accession AHIT00000000 [95]
*Lactobacillus rossiae* DSM 15814, sequence accession AKZK00000000 [96]
*Lactococcus garvieae* IPLA 31405, sequence accession AKFO00000000 [94]
*Paenibacillus polymyxa* OSY-DF, sequence accession AIPP00000000 [97]
*Pediococcus pentosaceus* strain IE-3, sequence accession CAHU01000001 through CAHU01000091 [98]
*Pelosinus fermentans* A11, sequence accession AKVM00000000 [99]
*Pelosinus fermentans* B4, sequence accession AKVJ00000000 [99]
*Pelosinus fermentans* JBW45, sequence accession AKVO00000000 [100]
*Pelosinus fermentans* R7, sequence accession AKVN00000000 [101]
*Planococcus antarcticus* DSM 14505, sequence accession AJYB00000000 [102]
*Rhodococcus sp.* strain DK17, sequence accession AJLQ00000000 [104]
*Staphylococcus aureus* Strain LCT-SA112, sequence accession AJLP00000000 [105]
*Staphylococcus capitis* QN1, sequence accession AJTG00000000 [106]
*Staphylococcus equorum subsp. equorum* Mu2, sequence accession CAJL01000001 to CAJL01000030 [107]
*Staphylococcus hominis* ZBW5*,* sequence accession AKGC00000000 [108]
*Staphylococcus saprophyticus subsp. saprophyticus* M1-1, sequence accession AHKB00000000 [109]
*Streptococcus mutans* GS-5, sequence accession CP003686 [110]
*Streptococcus pyogenes* M1 476, sequence accession AP012491 [111]
*Streptococcus salivarius* PS4, sequence accession AJFW00000000 [112]
*Streptococcus thermophilus* Strain MN-ZLW-002, sequence accession CP003499 [113]
*Ureibacillus thermosphaericus* Strain Thermo-BF, sequence accession AJIK00000000 [114]

### Phylum Tenericutes

*Mycoplasma leachii* Strain PG50^T^, sequence accession CP002108.1 [115]
*Mycoplasma mycoides subsp. mycoides*, sequence accession CP002107.1 [115]
*Mycoplasma wenyonii* Strain Massachusetts, sequence accession CP003703 [116]

### Phylum Actinobacteria

*Actinomyces massiliensis* Strain 4401292^T^, sequence accession AKIO00000000 [117]
*Bifidobacterium animalis subsp. lactis* B420, sequence accesion CP003497 [118]
*Bifidobacterium animalis subsp. lactis* *Bi-07,* sequence accesion CP003498 [118]
*Bifidobacterium bifidum* strain BGN4, sequence accession CP001361 [119]
*Brevibacterium massiliense* Strain 541308^T^, sequence accession CAJD00000000 [120]
*Corynebacterium bovis* DSM 20582, sequence accession AENJ00000000 [121]
*Corynebacterium diphtheriae* Biovar Intermedius NCTC 5011, sequence accession AJVH00000000 [122]
*Corynebacterium pseudotuberculosis* strain 1/06-A, sequence accession CP003082 [123]
*Corynebacterium pseudotuberculosis* strain 3/99-5 sequence accession CP003152.1 [124]
*Corynebacterium pseudotuberculosis* strain 42/02-A, sequence accession CP003062 [124]
*Microbacterium yannicii*, sequence accession CAJF01000001 through CAJF01000067 [125]
*Micromonospora lupini* Lupac 08, sequence accession CAIE01000001 [126]
*Mycobacterium bolletii* Strain M24, sequence accession AJLY00000000 [127]
*Mycobacterium intracellulare* Clinical Strain MOTT-36Y, sequence accession CP003491 [128]
*Mycobacterium massiliense* M18, sequence accession AJSC00000000 [129]
*Mycobacterium massiliense* strain GO 06, sequence accession CP003699 [130]
*Mycobacterium massiliense* strain M154, sequence accession AJMA00000000 [131]
*Mycobacterium tuberculosis* RGTB327, sequence accession CP003233 [132]
*Mycobacterium tuberculosis* MTB423, sequence accession CP003234 [132]
*Parascardovia denticolens* IPLA 20019, sequence accession AKII00000000 [133]
*Saccharothrix espanaensis* DSM 44229^T^, sequence accession HE804045 [134]
*Streptomyces auratus* Strain AGR0001, sequence accession AJGV00000000 [135]
“*Streptomyces cattleya*” DSM46488^T^, sequence accession FQ859185 and FQ859184 [136]
*Streptomyces globisporus* C-1027, sequence accession AJUO00000000 [137]
*Streptococcus mutans* GS-5, sequence accession CP003686 [138]
*Streptomyces sp.* Strain AA1529, sequence accession ALAP00000000 [139]
*Streptomyces sulphureus* L180, sequence accession AJTQ0000000 [140]

### Phylum Spirochaetes

*Borrelia crocidurae*, sequence accession CP003426 (chromosome), CP003427 to CP003465 (plasmids) [141]
*Treponema sp.* Strain JC4, sequence accession JQ783348 [142]

### Phylum Bacteroidetes

*Flavobacterium sp.* Strain F52, sequence accession AKZQ00000000 [143]
*Fusobacterium nucleatum subsp. fusiforme* ATCC 51190^T^, sequence accession AKXI00000000 [144]
*“Imtechella halotolerans”* K1^T^, sequence accession AJJU00000000 [145]

## Virus genomes

### Bacteriophage

Actinophage PIS136, sequence accession JX006077 [146]
Aeromonas hydrophila Phage CC2, sequence accession JX123262 [147]
Bacteriophage BC-611, sequence accession AB712291 [148]
Bacteriophage SSU5, sequence accession JQ965645 [149]
Blattabacterium sp. strain BGIGA, sequence accession [150]
Caulobacter crescentus Bacteriophage φCbK, sequence accession JX163858 [151]
*Celeribacter* Bacteriophage P12053L, sequence accession JQ809650 [152]
Croceibacter Bacteriophage P2559S, sequence accession JQ867099 [153]
Cronobacter sakazakii Temperate Bacteriophage phiES15 JQ780327 [154]
Marinomonas Bacteriophage P12026, sequence accession JQ867100 [155]
Pectobacterium carotovorum subsp. carotovorum Bacteriophage PP1, sequence accession JQ837901 [156]
Persicivirga bacteriophages P12024L, sequence accession JQ823123 [157]
Persicivirga bacteriophages P12024S, sequence accession JQ823122 [157]
phage clP1, sequence accession JN051154 [158]
Pseudomonas aeruginosa Siphophage MP1412, sequence accession JX131330 [159]
*Pseudomonas aeruginosa* Temperate Phage MP29, sequence accession EU272036 [160]
*Pseudomonas aeruginosa* Temperate Phage MP42, sequence accession JQ762257 [160]
Pseudomonas Phage Φ-S1, sequence accession JX173487 [161]
Siphophage MP1412, sequence accession JX131330 [162]
Staphylococcus aureus Bacteriophage GH15, sequence accession JQ686190 [163]
Vibrio vulnificus Bacteriophage SSP002, sequence accession JQ692107 [164]

### Eukaryotic viruses

African bovine rotaviruses RVA/Cow-wt/ZAF/1603/2007/G6P, sequence accession S9(VP7) JN831209, S4(VP4) JN831210, S6(VP6) JN831211, S1(VP1) JN831212, S2(VP2) JN831213, S3(VP3) JN831214, S5(NSP1) JN831204, S8(NSP2) JN831205, S7(NSP3) JN831206, S10(NSP4) JN831207, S11(NSP5) JN831208 [165]
African bovine rotaviruses RVA/Cow-wt/ZAF/1604/2007/G8P, sequence accession S9(VP7) JN831220, S4(VP4) JN831221, S6(VP6) JN831222, S1(VP1) JN831223, S2(VP2) JN831224, S3(VP3) JN831225, S5(NSP1) JN831215, S8(NSP2) JN831216, S7(NSP3) JN831217, S10(NSP4) JN831218, S11(NSP5) JN831219 [165]
African bovine rotaviruses RVA/Cow-wt/ZAF/1605/2007/G6P, sequence accession S9(VP7) JN831231, S4(VP4) JN831232, S6(VP6) JN831233, S1(VP1) JN831234, S2(VP2) JN831235, S3(VP3) JN831236, S5(NSP1) JN831226, S8(NSP2) JN831227, S7(NSP3) JN831228, S10(NSP4) JN831229, S11(NSP5) JN831230 [165]
Avian Leukosis Virus, sequence accession JX254901 [166]
Avian Influenza Virus H3N2, sequence accession JX175250 through JX175257 [167]
Avian Influenza Virus H5N2, sequence accession JQ990145 through JQ990152 [168]
Avian-Like H4N8 Swine Influenza, sequence accession JX151007 through JX151014 [169]
Avian Paramyxovirus, sequence accession JQ886184 [170]
Avian Tembusu-Related Virus Strain WR, sequence accession JX196334 [171]
Bluetongue Virus Serotype 9, sequence accession JX003687 to JX003696 [172]
Bluetongue Virus Serotype 16, sequence accession [173]
Bombyx mori Nucleopolyhedrovirus, sequence accession JQ991009 [174]
Bovine Viral Diarrhea Virus 2, sequence accession JF714967 [175]
Bovine Foamy Viruses, sequence accession JX307861 [176]
Canine Noroviruses, sequence accession FJ692500 and FJ692501 [177]
Chicken Anemia Virus, sequence accession JX260426 [178]
Chikungunya Virus, sequence accession JX088705 [179]
Chinese Virulent Avian Coronavirus GX-YL5, sequence accession HQ848267 [180]
Chinese Virulent Avian Coronavirus GX-YL9, sequence accession HQ850618 [180]
Coxsackievirus B4, sequence accession JX308222 [181]
Enterovirus C (HEV-C117), sequence accession JX262382 [182]
Genotype 4 Hepatitis E Virus Strain, sequence accession JQ993308 [183]
H10N8 Avian Influenza Virus, sequence accession JQ924786 to JQ924793 [184]
H9N2 Subtype Influenza Virus FJG9, sequence accession JF715008.1, JN869514.1 through JN869520.1 [185].
Herpes Simplex Virus 1 Strain McKrae, sequence accession JX142173 [186]
Human Coronavirus NL63, sequence accession JX104161 [187]
Human G10P Rotavirus, sequence accession AB714258 through AB714268 [188]
Ikoma Lyssavirus, sequence accession JX193798 [189]
Korean sacbrood viruses AmSBV-Kor19, sequence accession JQ390592 [190]
Korean sacbrood viruses AmSBV-Kor21, sequence accession JQ390591 [190]
Mitochondrion of Frankliniella occidentalis*, sequence accession* JN835456 [191]
New Circular DNA Virus from Grapevine, sequence accession JQ901105 [192]
Novel Porcine Epidemic Diarrhea Virus, sequence accession JX112709 [193]
Pararetrovirus, sequence accession JQ926983 [194]
Parechovirus, sequence accession JX050181 [195]
Peste des Petits Ruminants Virus, sequence accession JX217850 [196]
Polyomavirus, sequence accession JQ412134 [197]
Porcine Circovirus 2b Strain CC1, sequence accession JQ955679 [198]
Porcine circovirus type 2 (PCV2), sequence accession JX294717 [199]
Porcine Epidemic Diarrhea Virus Strain AJ1102, sequence accession JX188454 [200]
Porcine Sapelovirus Strain YC2011, sequence accession JX286666 [201]
Respiratory Syndrome Virus Strain QY2010, sequence accession JQ743666 [202]
SAT 2 Foot-and-Mouth Disease Virus, sequence accession JX014255 [203]
SAT 2 Foot-and-Mouth Disease Virus PAT, sequence accession JX014256 [203]
Street Rabies Virus, sequence accession HQ450386 [204]
Waterfowl aviadenovirus goose adenovirus 4, sequence accession JF510462 [205]

## Plant genomes

cpDNA of Smilax china*, sequence accession* NC_015104 [206]
Elodea canadensis*, sequence accession* JQ310743 [207]
Ogura-type mitochondrial genome, sequence accession AB694743 [208]

## Fungal genomes

*Aspergillus oryzae* Strain 3.042, sequence accession AKHY00000000 [209]
*Rhodosporidium toruloides* MTCC 457, sequence accession AJMJ00000000 [210]

## Animal genomes

*Helicoverpa armigera*, sequence accession HQ613271 [211]

## Plasmids

plasmidIncN plasmid pRSB201, sequence accession JN102341 [212]
plasmidIncN plasmid pRSB203, sequence accession JN102342 [212]
plasmidIncN plasmid pRSB205, sequence accession JN102343 [212]
plasmidIncN plasmid pRSB206, sequence accession JN102344 [212]
